# Multiple verrucous squamous cell carcinomas developing on chronic hidradenitis suppurativa lesions—a rare case report from Romania

**DOI:** 10.3389/fmed.2024.1336688

**Published:** 2024-01-12

**Authors:** Gyula László Fekete, László Fekete, László Barna Iantovics, Júlia E. Fekete, Ilarie Brihan

**Affiliations:** ^1^Department of Dermatology, Dermatology Clinic, George Emil Palade University of Medicine, Pharmacy, Science, and Technology, Târgu Mureș, Romania; ^2^CMI Dermamed Private Medical Office, Târgu Mureș, Romania; ^3^Doctoral School, George Emil Palade University of Medicine, Pharmacy, Science, and Technology, Târgu Mureș, Romania; ^4^Department of Electrical Engineering and Information Technology, George Emil Palade University of Medicine, Pharmacy, Science and Technology, Târgu Mureș, Romania; ^5^Regional Center for Public Health, National Institute of Public Health, Târgu Mureș, Romania; ^6^Department of Dermatology, Dermatology Clinic, Faculty of Medicine and Pharmacy, University of Oradea, Oradea, Romania

**Keywords:** hidradenitis suppurativa, squamous cell carcinoma, verrucous carcinoma, inflammatory lesions, comorbidities

## Abstract

Hidradenitis suppurativa (HS) is an uncommon, recurrent, inflammatory skin illness of the apocrine glands, with a questionable etiology. The disease is associated with a multitude of comorbidities, of which the appearance of malignancy is the most important. Squamous cell carcinoma is considered the most frequent malignancy that can appear in HS. A case report of a 72 years-old male is presented, who suffered over 40 years from persistent, extensive hidradenitis suppurativa in stage Hurley III, on the buttocks and perianal region, who recently presented two verrucous semi-consistent, skin-colored tumors on the right buttock. The biopsy and histopathological exam confirmed a verrucous type of squamous cell carcinoma. There are about 100 reported clinical cases of squamous cell carcinoma complicating hidradenitis suppurativa in the literature, but only a few describe a verrucous carcinoma as a clinical form. The particularity of the case is the rare appearance of multiple verrucous types of squamous cell carcinomas in a male patient, in Hurley Stage III, with a long HS disease duration, appearing on the perianal/gluteal region, being the first case report in our country. We suggest that a tumor screening should be done for all the patients with HS who have these risks.

## 1 Introduction

Hidradenitis suppurativa is a chronic inflammatory disease, with the appearance of follicular obstructions, abscesses, fistulas, foul mucopurulent secretions, and vicious, often debilitating scarring of the apocrine gland areas of the skin (1). The average prevalence in Europe of the disease is about 1% (2). The first case was published in 1839 by Valpeau (3). The disease was described by Verneuil (3), mentioning that the chronic inflammatory lesions typically involve the folds and the buttock. The appearance of the clinical features can develop symptoms like pain, pruritus, and debilitating scars, with implications for the quality of life (4). The disease is at the border of different specialties like dermatology, general surgery, or plastic surgery and for this reason, a precise diagnosis is generally delayed (5). In the evolution of HS, a large number of associated diseases can occur. From these, the appearance of malignancies is the most important. In a recent review, Gierek et al. (6) highlighted that in the evolution of HS, the appearance of nonmelanoma skin cancer (NMSC), hematologic malignancies, and metastatic cancers are possible. From these, the most frequent complication is the development of squamous cell carcinoma (SCC). Also from this review, the authors analyzed 74 cases of SCC that appeared on HS, and they concluded that the majority of the primary squamous cell carcinomas were an ulcerated or nodule-type form (6). There are about 100 reported cases of squamous cell carcinoma complicating hidradenitis suppurativa in the literature, but only a few describe a verrucous carcinoma type as a clinical form (6). We present a clinical case of verrucous squamous cell carcinoma developing on chronic HS.

## 2 Case report

**Figure 1:**
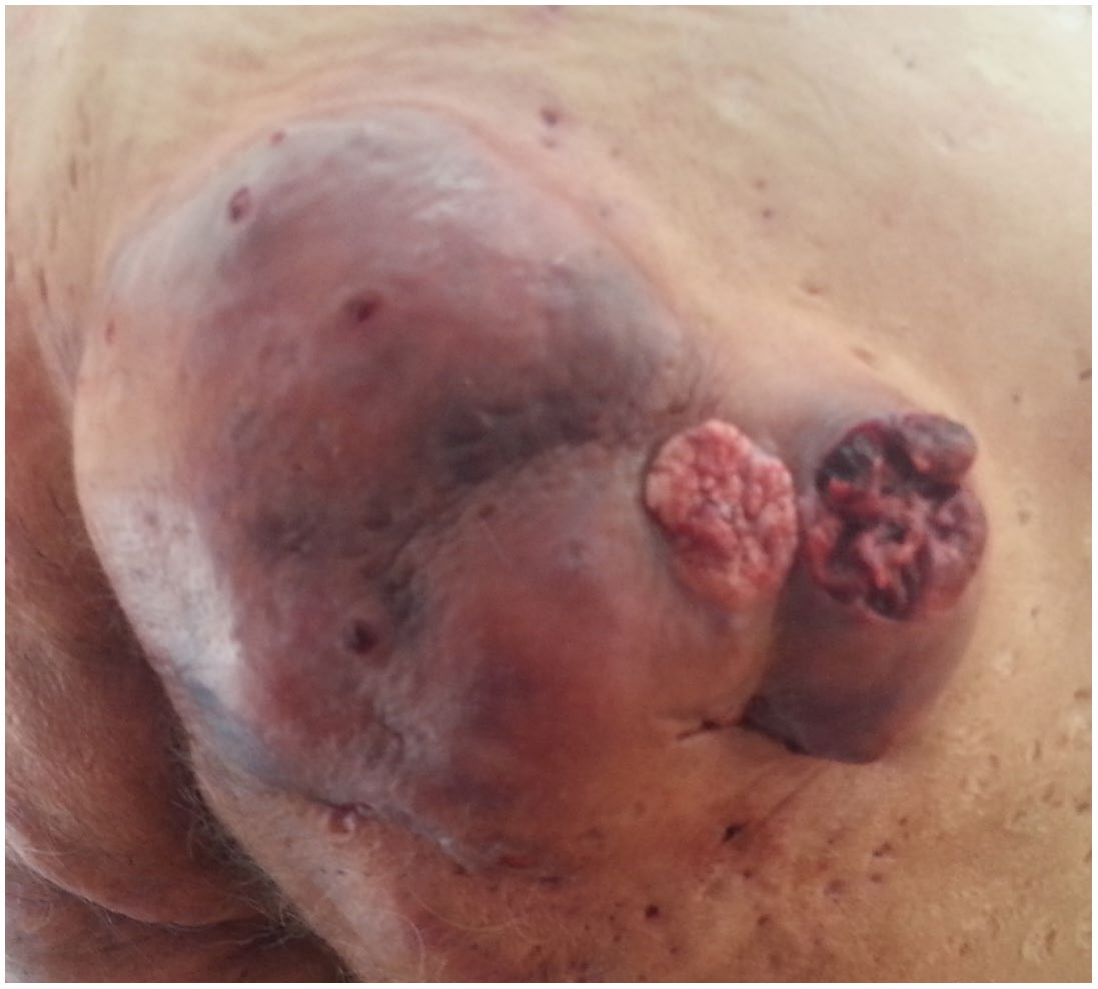
Clinical aspect of the tumors on the right buttock.

**Figure 2:**
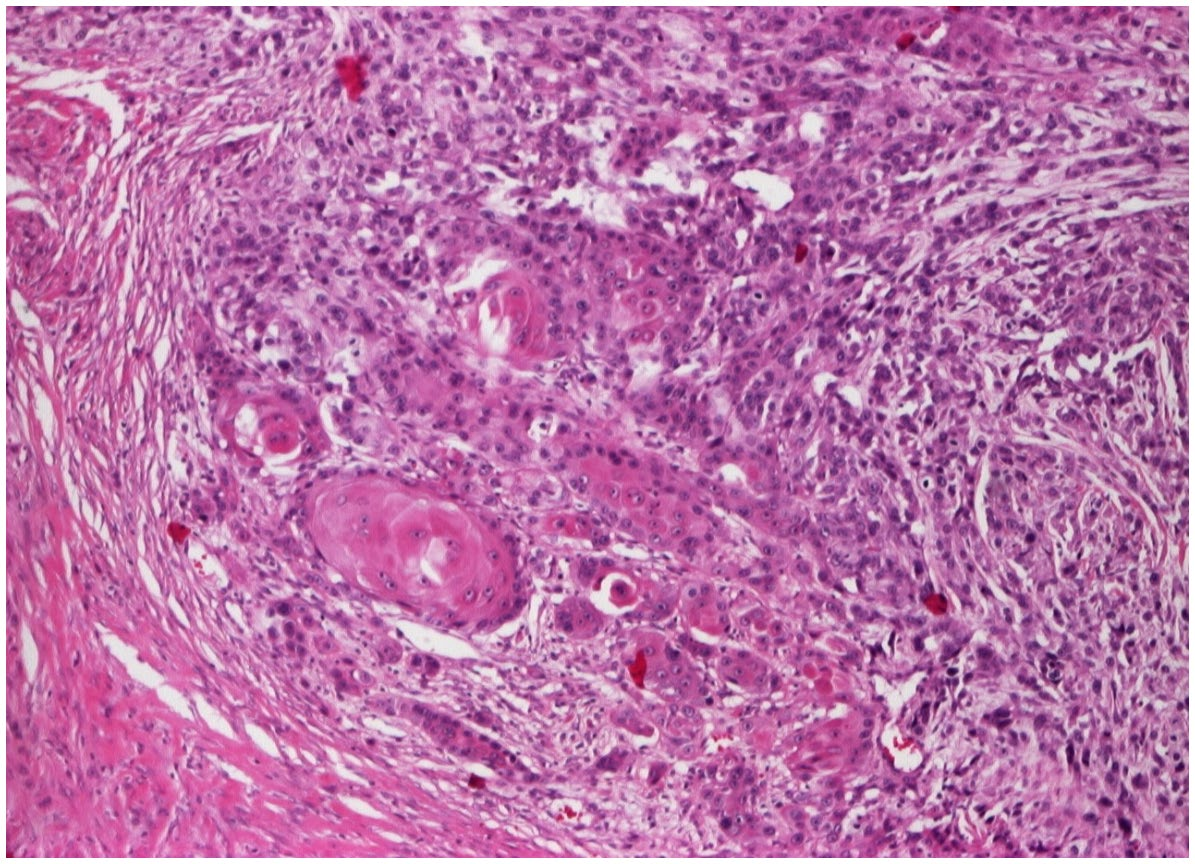
Biopsy, buttocks (H&E 10×): poorly differentiated verrucous squamous cell carcinoma with marked cytonuclear pleomorphism and atypical mitoses infiltrating into the dermis.

We present a clinical case of a 72 years-old, non-smoking, immune-competent normoponderal patient, who suffered over 40 years from persistent, extensive hidradenitis suppurativa on the buttocks and perianal region. He was treated for over 40 years with oral antibiotics and retinoids, local topical antibiotics, steroids, and a multitude of antiseptics without success. He had periods of remission and exacerbation. During the dermatological consultation, we found an active area of HS in stage Hurley III on the buttocks and perianal region and two verrucous semi-consistent, skin-colored tumors on the right buttock, having a base diameter of 2.5 and 3 cm, presenting spontaneous bleeding (Figure 1). These tumors developed relatively quickly in approximately 3 months. The patient is suffering from several chronic diseases, like chronic obstructive pulmonary disease, essential hypertonia, arthrosis, and osteoporosis, which were under medical control and are not related to HS. He is not suffering from diabetes. His family medical history was unremarkable. The results of routine laboratory testing like hematology and biochemistry were within the normal limits. The treatment decision was the surgical removal of the tumors. The histopathological examination of the two excised tumors confirmed the verrucous type of squamous cell carcinoma (Figure 2). Based on the clinical and histological examination, the patient was transferred to an oncology service for further examination and treatment options. Unfortunately, due to the advanced stage of the carcinoma, the evolution was fatal for the patient.

## 3 Discussion

Hidradenitis suppurativa (HS) is a chronic and exhausting dermatologic disease of the apocrine glands, characterized by the formation of multiple inflammatory lesions, abscesses, fistulas, and scars, especially arising on folds and buttocks. The exact prevalence of HS is unknown, it has been estimated to be as high as 4.1%, and it is three times more frequent in women than in men (7). The etiology is still unclear, but the illness is frequently associated with smoking, poor hygiene, immunocompromised status, and diseases like metabolic, cardiovascular, endocrine, gastrointestinal, rheumatologic, and psychiatric ones, and also with reduced cutaneous levels of calprotectin, zinc, or ascorbate, which all together compromise the life quality of these patients (4). The clinical aspect of HS is multiform, from inflammatory lesions to nodules, abscesses, fistulas, and scars that can be present, as is well defined by the Hurley staging. The differential diagnoses can include bacterial, especially Staphylococcal skin infections, with or without elementary lesions like abscesses, carbuncles, and furuncles, and cutaneous Crohn’s disease. Also, different types of cysts, like Bartholin or epidermoid cysts can resemble HS (8). Squamous cell carcinoma (SCC) is the second most common skin cancer, accounting for 20% of skin cancers (9). Squamous cell carcinomas comprise different types of cancers that are formed on the surface of the skin and mucous membranes (10). The most important general risk factors for the development of SCC on the skin, are sun exposure, age, and phototype of the skin, especially in cases of phototype I–III was described the mutation in the suppressor protein (TP53) (11). Squamous cell carcinomas may arise in a multitude of chronic inflammatory dermatoses, wounds, and scars such as thermal burn scars, discoid lupus erythematosus, chronic ulcerations, chronic radiodermatitis, precancers, etc. (12–19). In the literature, we have found published cases of SCC as a severe complication of chronic HS lesions. The relationship between HS lesions and SCC is poorly understood and must be further explored (20). The development of SCC in cases of HS, is multifactorial. HS is more prevalent in women, but the appearance of SCC on the disease is more frequent in men (6). Immunosuppression due to chronic disease is one of the risk factors. Also, the location of HS lesions is important, because most of the published cases of SCC on HS lesions are located in the gluteal and perianal region (21–26). Gierek et al. (6), found that 94.59% of the cases of SCC were developed in the perianal/gluteal region of HS. Most of the reported cases were in Hurley stage III of the disease and were males, like in our presented case (25, 27–29). Also, Gierek et al. (6), concluded that the average age of the patient with SCC in HS lesions was 52.6 years, and the mean time from onset SCC was 25.79 years (range 8 years to 53 years), and most of the patients were in Hurley Stage III (97.2%). In two articles the authors suggest the presence of the HPV virus as a causative factor for the appearance of SCC in HS (30, 31). We cannot prove this hypothesis in our case. The studied references present mostly ulcerated, nodular, and metastatic clinical forms of SCC developed from HS. Cosman et al. (32), present a paper about a verrucous form, mentioning that their case is the second published with this clinical form, like in our case presentation. Also, we did not find any references about the presence of two SCC tumors at the same time in a patient with HS. New ultrasound techniques are used to diagnose the possible transformation of HS into squamous cell carcinoma. Wortsman (33) describes the ultrasound diagnostic criteria for HS and suggests new scores for the severity of the disease. Zussino et al. (34) describe the usefulness of color Doppler in the staging and the follow-up of the evolution of HS. Nazzaro et al. (35) comparing the clinical forms and sonographic scores in hidradenitis suppurativa, propose a new ultrasound scoring system for the follow-up on the evolution of HS, including malignant transformation. Treatment of HS is difficult due to a lack of effective medical therapies. Modern possibilities, like biological therapies, open a new era in the treatment of this disease (36). The treatment of this complication is surgical and depending on the staging of the tumor, is oncologic. Considering the high mortality rate in these cases, we suggest screening for an early diagnosis for the possibility of the appearance of SCC lesions in all HS patients (37).

## 4 Conclusion

The particularity of the case is the rare appearance of multiple verrucous types of squamous cell carcinomas on a chronic, recurrent, inflammatory dermatologic disease like HS, which is the first case published in our country. The potential risk factors such as sex, advanced stage, chronic evolution, and specific localization as perianal/gluteal and buttock region should be considered in the malignant transformation of HS. We recommend a dermatological cancer screening to all patients with HS who have these risks.

## Data availability statement

The raw data supporting the conclusions of this article will be made available by the authors, without undue reservation.

## Ethics statement

The studies involving humans were approved by the Dermamed Private Office Ethics Committee. The studies were conducted in accordance with the local legislation and institutional requirements. The participants provided their written informed consent to participate in this study. Written informed consent was obtained from the individual(s) for the publication of any potentially identifiable images or data included in this article.

## Author contributions

GF: Conceptualization, Formal analysis, Funding acquisition, Investigation, Methodology, Resources, Supervision, Validation, Visualization, Writing – original draft, Writing – review & editing, Project administration. LF: Conceptualization, Formal analysis, Investigation, Methodology, Resources, Validation, Visualization, Writing – original draft, Writing – review & editing. LI: Visualization, Writing – review & editing, Data curation, Software. JF: Conceptualization, Formal analysis, Investigation, Methodology, Validation, Visualization, Writing – original draft, Writing – review & editing. IB: Conceptualization, Formal analysis, Investigation, Methodology, Validation, Writing – original draft, Writing – review & editing.
